# The Inflammatory-Immune Axis in Thyroid Disease: A Mendelian Randomization Study

**DOI:** 10.1155/ije/6644708

**Published:** 2025-07-21

**Authors:** Tao Pan, Zhihao Fang, Titi Hui, Xuanlin Wu, Xu Hu, Jiabin You, Chang Liu

**Affiliations:** Department of General Surgery, Fourth Affiliated Hospital of Harbin Medical University, Harbin, China

**Keywords:** Graves' disease, Hashimoto's thyroid, inflammatory cytokines, Mendelian randomization, thyroid cancer

## Abstract

**Background:** An increasing body of research has highlighted a close association between circulating inflammatory proteins and thyroid diseases. However, whether this relationship is causal or if immune cells act as intermediaries remains uncertain.

**Methods:** In this study, we conducted a bidirectional two-sample Mendelian randomization (MR) analysis using data from genome-wide association studies (GWAS) to investigate the potential causal relationships between circulating inflammatory cytokines/proteins, immune cells, and three thyroid diseases: Graves' disease (GD), Hashimoto's thyroiditis (HT), and thyroid cancer (TC). We conducted MR analysis using five methods, with the inverse variance-weighted (IVW) approach as the primary method. Sensitivity analyses were performed to assess horizontal pleiotropy and heterogeneity. To enhance result reliability, we applied a False Discovery Rate (FDR) correction to control for multiple testing biases. Lastly, we utilized a two-step MR design to explore the potential mediating role of immune cells in these causal relationships.

**Results:** Our findings demonstrated a negative association between CCL19 and GD, suggesting that higher levels of CCL19 may be associated with a lower risk of developing GD. Additionally, CCL19 showed a positive correlation with FSC-A on CD4+ T cells, indicating that elevated CCL19 levels are linked to larger cell sizes (FSC-A) in CD4+ T cells. Moreover, FSC-A on CD4+ T cells was inversely associated with GD, suggesting that larger CD4+ T cells (with higher FSC-A) may be linked to a reduced risk of GD. These results indicate that immune cells may act as intermediaries in the pathway involving circulating inflammatory proteins and GD.

**Conclusion:** The study establishes a causal relationship between circulating inflammatory proteins and immune cells in relation to GD, with immune cells serving as intermediaries in the pathway between inflammatory proteins and GD risk.

## 1. Introduction

Thyroid diseases represent a prevalent clinical condition, encompassing both autoimmune thyroid diseases (AITDs) and thyroid cancer (TC) [1]. Hashimoto's thyroiditis (HT) and Graves' disease (GD) are the most frequently diagnosed forms of AITDs. Thyroid cancer occurs in patients with AITDs, with a predilection for those with HT [2, 3]. Epidemiological studies have demonstrated that in iodine-sufficient regions, HT is the predominant cause of hypothyroidism, whereas GD serves as the leading cause of hyperthyroidism [4–6]. Hypothyroidism constitutes a significant risk factor for cardiovascular disease [7] and has diverse effects on the nervous, musculoskeletal, and gastrointestinal systems [8]. Hyperthyroidism is linked to conditions such as atrial fibrillation, stroke, pulmonary embolism, and a hypercoagulable state [9]. TC is the most prevalent malignant endocrine tumor. Epidemiological data reveal that in 2020, there were 586,000 reported cases of thyroid cancer worldwide, ranking it ninth in global incidence [10]. Thyroid diseases impose a substantial economic burden on society. At present, their pathogenesis remains under investigation, with HT believed to be primarily mediated by Th1 cells targeting thyroid peroxidase and thyroglobulin antigens, while GD is mainly mediated by Th2 cells targeting the TSH receptor [11, 12]. The treatment of thyroid diseases remains suboptimal, with GD management primarily relying on antithyroid drugs, radioactive iodine, and surgery, all of which carry risks of relapse, hypothyroidism, and complication [13]. HT is mainly treated with thyroid hormone replacement, though the efficacy is limited and thyroid function can fluctuate [14]. Treatment for TC includes surgery, radioactive iodine, and targeted therapy; however, its efficacy in advanced stages is limited, with high side effects and costs [15]. There remains a pressing need to explore more precise and individualized treatment strategies that can reduce side effects and improve efficacy, particularly for refractory and advanced patients, to achieve better disease control and long-term management.

Inflammation is a physiological response of the host to infection or injury. However, aberrant inflammatory responses can lead to tissue damage and are central to the pathogenesis of various diseases, including autoimmune disorders and cancer. It has been observed that several inflammatory cytokines/proteins exhibit abnormal levels in thyroid diseases [16–18]. There appears to be a close association between inflammatory cytokines, immune cells, and thyroid diseases. Nevertheless, these studies have predominantly been observational, with inherent limitations such as confounding variables and reverse causality, making it challenging to establish a causal relationship.

Mendelian randomization (MR) is based on Mendel's laws of inheritance, leveraging the random allocation of genes to minimize confounding factors and reverse causality, thus offering more robust causal inferences [19]. Our objective was to elucidate the causal relationships and directions between inflammatory cytokines/proteins and thyroid diseases. Additionally, we aimed to determine whether immune cells act as mediators in the pathway from inflammatory proteins to thyroid diseases. This will deepen our understanding of the role of these inflammatory factors/proteins and immune cells in the pathogenesis of thyroid diseases. This knowledge could facilitate the development of personalized therapeutic strategies, enabling more precise and effective management of thyroid diseases.

## 2. Materials and Methods

### 2.1. Study Design

**Figure 1:**
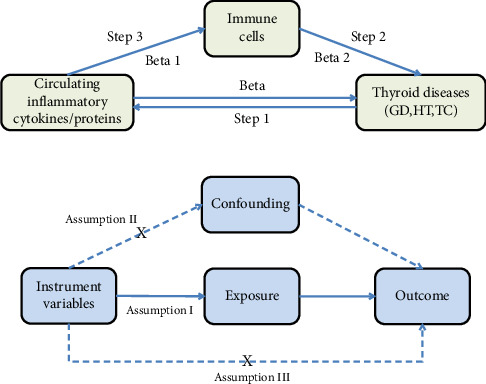
The flowchart of the study.

The MR study was conducted to evaluate the causal relationships between 41 circulating inflammatory cytokines and 91 circulating inflammatory proteins with three thyroid diseases (GD, HT, and TC). The analysis was conducted in three sequential stages, as illustrated in Figure 1. First, the causal effects of 41 inflammatory cytokines and 91 inflammatory proteins on the three thyroid diseases were examined (Step 1). Subsequently, the causal effects of 731 immune cell types on the three thyroid diseases were assessed (Step 2). Finally, the immune cell-mediated effects of circulating inflammatory proteins on the pathogenic pathways of thyroid diseases were investigated (Step 3). The total effect of circulating inflammatory cytokines/proteins on thyroid diseases is represented by beta. The direct effect of circulating inflammatory cytokines/proteins on immune cells is denoted as beta1, while the direct effect of immune cells on thyroid diseases is indicated by beta2. The “product of coefficients” method was employed to calculate the Indirect Effect mediated by immune cells in the pathway between circulating inflammatory cytokines/proteins and thyroid diseases (beta1 × beta2), as well as the Mediated Proportion ([beta1 × beta2]/beta). In this MR study, single nucleotide polymorphisms (SNPs) were employed as instrumental variables (IVs), and three key assumptions were satisfied: The genetic instrument (SNP) must be strongly associated with the exposure (relevance assumption), must be independent of any confounders that might influence the outcome (independence assumption), and should affect the outcome only through the exposure, without any other pathways (exclusion restriction assumption). Additionally, this MR study adheres to the STROBE-MR guidelines [20]. All original studies received ethical approval and informed consent, and no additional ethical approval was necessary for our study.

### 2.2. Data Source

The dataset for the 41 circulating inflammatory cytokines was derived from a GWAS meta-analysis, integrating results from the Young Finns Study and the FINRISK survey, encompassing 8293 Finnish individuals. The GWAS data can be accessed at https://data.bris.ac.uk/data/dataset/3g3i5smgghp0s2uvm1doflkx9x. Summary statistics for the GWAS data on 91 circulating inflammatory proteins are publicly available in the EBI GWAS Catalog, under accession numbers GCST90274758 to GCST90274848. These data encompass 11 study cohorts comprising 14,824 participants of European ancestry. The GWAS summary statistics for immune cells were retrieved from the GWAS catalog (accession numbers GCST0001391 to GCST0002121) [21]. This GWAS involved 3757 nonoverlapping individuals of European descent. The SNPs for these three thyroid diseases were sourced from the IEU database. The GWAS for GD included 1678 European cases and 456,942 European controls, comprising a total of 24,189,816 SNPs. The genetic data for HT comprised 15,654 European cases and 379,986 European controls, totaling 24,146,037 SNPs. The genetic data for TC included 1054 European cases and 490,920 European controls, amounting to 24,198,226 SNPs. The genetic data for GD, HT, and TC can be accessed respectively at: https://gwas.mrcieu.ac.uk/datasets/ebi-a-GCST90018847/, https://gwas.mrcieu.ac.uk/datasets/ebi-a-GCST90018855/, and https://gwas.mrcieu.ac.uk/datasets/ebi-a-GCST90018929/.

### 2.3. IVs Selection

Based on the three core assumptions of MR [1]: We initially set a stringent significance threshold of *p* < 5 × 10^−8^ to identify SNPs strongly associated with the three thyroid diseases and circulating inflammatory cytokines/proteins. However, due to the limited number of SNPs detected for TC and inflammatory cytokines/proteins under this criterion, we adjusted the threshold to *p* < 5 × 10^−6^. SNPs associated with immune cells were selected at a significance threshold of *p* < 1 × 10^−5^. For GD and HT as exposures, the SNP count was already adequate [2]. To ensure the independence of these IVs, we employed criteria for eliminating linkage disequilibrium (LD) set at kb = 10,000 and *r*^2^ = 0.001 [3]. Each significant exposure-related SNP was checked using the LDtrait function [22] in LD Link (https://ldlink.nci.nih.gov), and any SNPs potentially confounded by factors such as smoking and BMI were excluded. Due to the chronic inflammatory state associated with obesity, we also excluded SNPs potentially confounded by BMI for inflammatory cytokines/proteins. Additionally, palindromic SNPs were discarded, as we could not determine whether the directionality of these SNPs was consistent between the exposure and outcome [3]. We calculated the *R*^2^ value for each SNP to assess the proportion of variance in the exposure explained by the IV. Furthermore, we computed the F-statistic for each SNP to identify potential biases related to weak IVs, selecting only those with an F-statistic > 10 for further MR analysis. Supporting Tables 1–3 present comprehensive details of the significant IVs that were ultimately selected.

### 2.4. Statistical Analysis

We conducted the MR analysis using the “TwoSampleMR” package in R (version 4.3.2). The primary analytical approach was the inverse variance weighted (IVW) method. When no heterogeneity was detected, a fixed effects model for the IVW method was employed; otherwise, a random effects model was applied. To confirm the robustness of the causal estimates obtained from the IVW method, we employed a range of supplementary methods and conducted comprehensive sensitivity analyses. The supplementary methods included MR Egger [23], weighted median [24], weighted mode, and simple mode. For sensitivity analyses, Cochrane's Q test was used to assess heterogeneity, with a *p* value < 0.05 indicating the presence of heterogeneity [25]. Horizontal pleiotropy was evaluated using the intercept result from the MR Egger analysis, where a *p* value > 0.05 was considered as evidence against pleiotropy [23]. The MR-PRESSO method was employed to detect outliers and simultaneously test for pleiotropy [26]. A leave-one-out analysis was performed by iteratively excluding individual SNPs and conducting MR analysis on the remaining SNPs to determine if any single SNP had a disproportionate influence on the outcome [27]. Scatter plots and funnel plots were generated to further verify the robustness of the results. We used the MR-Steiger method to assess the accuracy of the presumed causal direction. When the nominal causal pairs' “correct_causal_direction” were flagged as “False,” it indicated that the genetic instrument's explanatory power for the outcome variable exceeded that for the exposure variable. This suggests that the hypothesized causal direction (exposure ⟶ outcome) might be incorrect, indicating a potential reverse causality, where the outcome might actually influence the exposure [28]. Additionally, The IVW results were adjusted using the False Discovery Rate (FDR) correction to control for the false positive rate in multiple hypothesis testing, thereby enhancing the accuracy of identifying causal relationships. An FDR-adjusted *p* value (pFDR) < 0.05 was considered indicative of a significant causal relationship. To identify potential immune cell characteristics mediating the relationship between inflammatory factors/proteins and the three thyroid diseases, a two-step MR analysis was performed to distinguish between the direct and indirect effects of immune cells and inflammatory factors/proteins. The first step examined the causal relationship of immune cells with the three thyroid diseases, identifying significant immune cell phenotypes as potential mediators. In the second step, we evaluated the influence of significant inflammatory factors/proteins on this mediator.

## 3. Results

### 3.1. Effect of the Circulating Inflammatory Cytokines/Proteins on Thyroid Diseases

**Figure 2:**
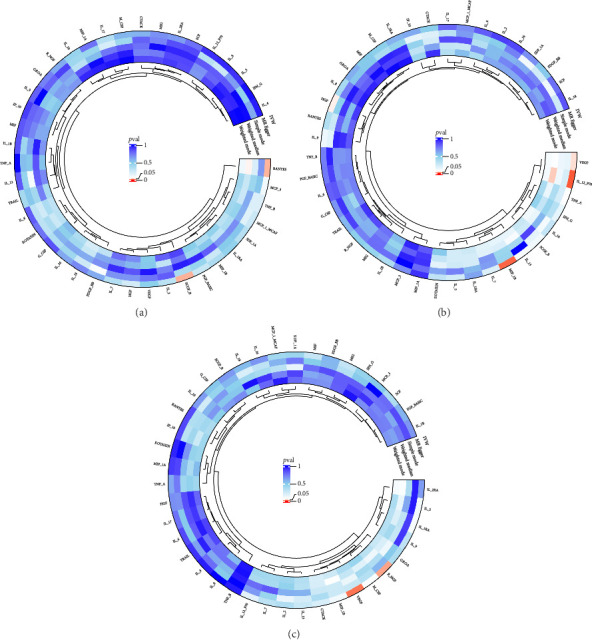
(a) Circular plot showing the causal relationship between 41 circulating inflammatory cytokines on GD. (b) Circular plot showing the causal relationship between 41 circulating inflammatory cytokines on HT. (c) Circular plot showing the causal relationship between 41 circulating inflammatory cytokines on TC.

**Figure 3:**
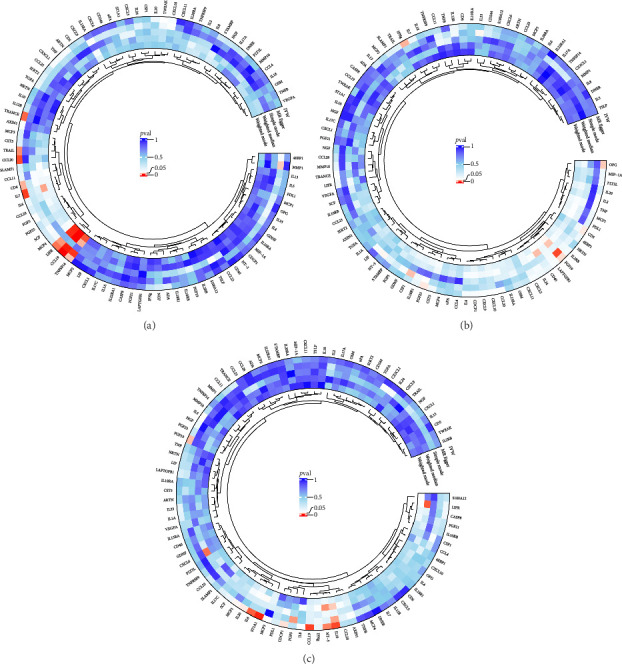
(a) Circular plot showing the causal relationship between 91 circulating inflammatory proteins on GD. (b) Circular plot showing the causal relationship between 91 circulating inflammatory proteins on HT. (c) Circular plot showing the causal relationship between 91 circulating inflammatory proteins on TC.

**Figure 4:**
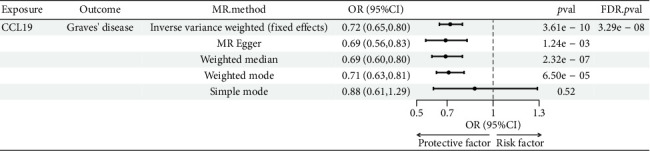
Forest plot showing the causal relationship between CCL19 on GD.

The preliminary IVW analysis identified nominal causal associations between specific circulating inflammatory cytokines and thyroid diseases. Among the 41 circulating inflammatory cytokines (Figures 2(a), 2(b), 2(c)), 2 were associated with GD, 4 were linked with HT, and 2 showed associations with TC (*p* < 0.05). Out of the 91 circulating inflammatory proteins (Figures 3(a), 3(b), 3(c)), 8 were nominally associated with GD, 5 with HT, and 10 with TC (*p* < 0.05). After applying the FDR correction, we found that only one of the 91 circulating inflammatory proteins, CCL19, exhibited a significant causal association with GD (pFDR < 0.05). The MR analysis results indicated a significant negative association between genetically predicted CCL19 and GD (OR: 0.72, 95% CI: 0.65–0.80, *p*=3.61E − 10, pFDR = 3.29E − 08, IVW) (Figure 4). Furthermore, the direction of the β estimates reflecting the causal relationship between these traits was consistent across all five MR methods, enhancing the robustness of our findings. More detailed MR analysis results are available in Supporting Table 4.

### 3.2. Effect of Thyroid Diseases on Circulating Inflammatory Cytokines/Proteins

**Figure 5:**
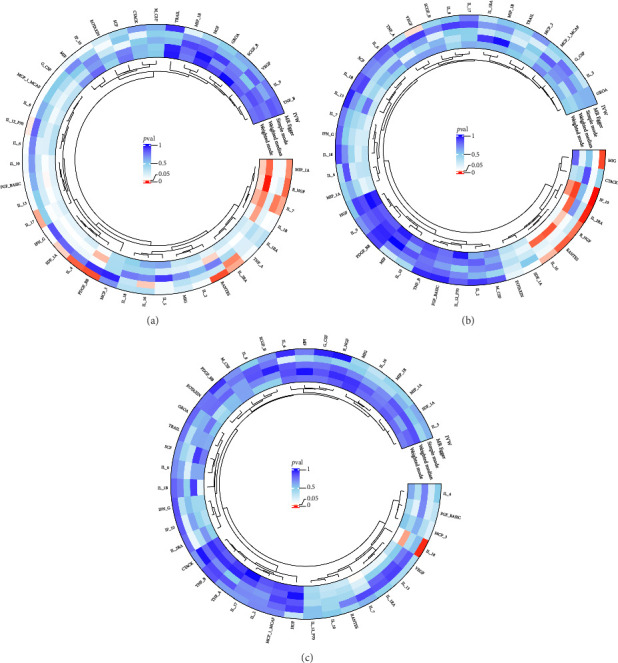
(a) Circular plot showing the causal relationship between GD on 41 circulating inflammatory cytokines. (b) Circular plot showing the causal relationship between HT on 41 circulating inflammatory cytokines. (c) Circular plot showing the causal relationship between TC on 41 circulating inflammatory cytokines.

**Figure 6:**
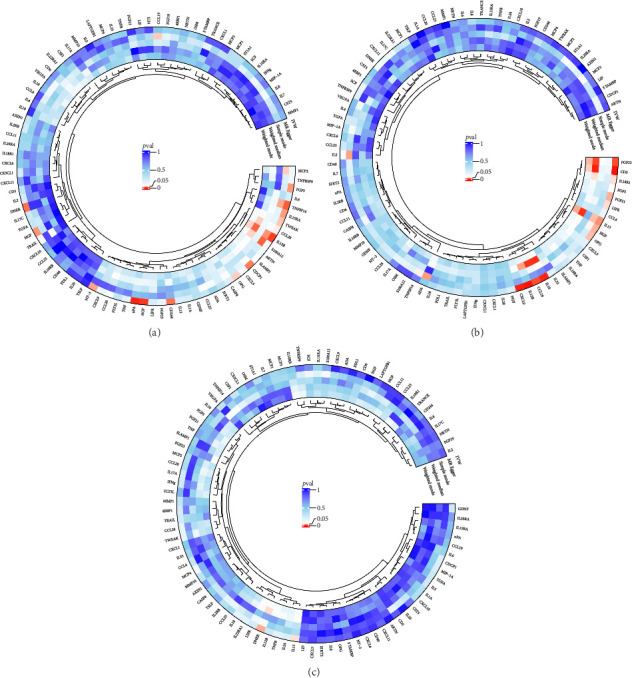
(a) Circular plot showing the causal relationship between GD on 91 circulating inflammatory proteins. (b) Circular plot showing the causal relationship between HT on 91 circulating inflammatory proteins. (c) Circular plot showing the causal relationship between TC on 91 circulating inflammatory proteins.

The preliminary IVW analysis revealed nominal causal associations in the MR analysis using 41 inflammatory cytokines as outcomes (Figures 5(a), 5(b), 5(c)). Specifically, GD showed associations with 10 inflammatory cytokines, HT with 7 inflammatory cytokines, and TC with 1 inflammatory cytokine (*p* < 0.05). In the MR analysis using 91 inflammatory proteins as outcomes (Figures 6(a), 6(b), 6(c)), nominal causal relationships were identified between GD and 14 inflammatory proteins, HT and 12 inflammatory proteins, and TC and 2 inflammatory proteins (*p* < 0.05). After excluding causal pairs with a “correct_causal_direction” marked as False and applying FDR correction to the *p* values, we found no evidence of causal associations between genetically predicted GD and circulating inflammatory cytokines/proteins. More comprehensive MR analysis results can be found in Supporting Table 5.

### 3.3. Mediation Analysis

**Figure 7:**
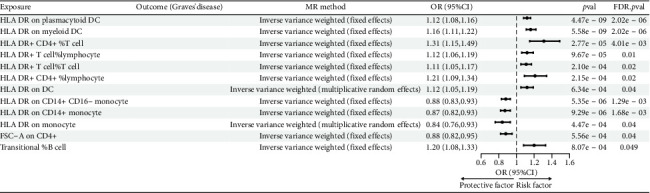
Forest plot showing the causal relationship between circulating immune cells on GD.

Given the significant causal relationship identified between CCL19 and GD, we proceeded with a two-sample MR analysis of immune cell phenotypes in relation to GD. After correcting for incorrect causal direction using the MR-Steiger method and adjusting for FDR, we identified 12 immune cell phenotypes that exhibited a significant causal association with GD (Figure 7). Genetically predicted HLA DR on plasmacytoid DC, HLA DR on myeloid DC, HLA DR+ CD4+ % T cell, HLA DR+ T cell% lymphocyte, HLA DR+ T cell% T cell, HLA DR+ CD4+ %lymphocyte, and HLA DR on DC showed a positive correlation with GD. Conversely, genetically predicted HLA DR on CD14+ CD16-monocyte, HLA DR on CD14+ monocyte, HLA DR on monocyte, Forward Scatter Area (FSC-A) on CD4+, and Transitional % B cell showed a negative association with GD (Supporting Table 6). Notably, there is evidence of horizontal pleiotropy in the causal relationship between HLA DR+ T cell % and GD (*p* < 0.05). The β estimates from IVW and MR-Egger analyses for Transitional % B cells and GD show inconsistent directions, prompting us to exclude these two causal relationships from further consideration. More detailed MR analysis results are provided in Supporting Table 7.

**Figure 8:**
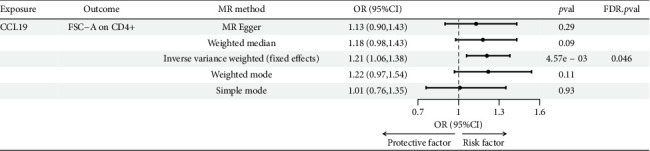
Forest plot showing the causal relationship between CCL19 on FSC-A on CD4+.

Following the fundamental principles of the two-step MR method for mediation analysis, we selected the aforementioned 12 immune cells as representative immune cells for subsequent mediation analysis. Subsequently, an MR analysis was performed between CCL19 and the 12 immune cells, and after FDR correction, the results indicated a significant positive association between the GD-related circulating inflammatory protein CCL19 and the GD-associated immune cell FSC-A on CD4+. Genetically predicted CCL19 showed a positive correlation with FSC-A on CD4+ (OR: 1.21; 95% CI: 1.06–1.38; *p*=4.57E − 03; pFDR = 0.046; IVW) (Figure 8). More detailed MR analysis results are provided in Supporting Table 8. Additionally, FSC-A on CD4+ accounted for 7.20% of the mediated proportion in the CCL19-GD pathway (Table 1). This suggests that immune cells act as mediator in the pathway involving circulating inflammatory proteins and GD.

### 3.4. Sensitivity Analysis

**Figure 9:**
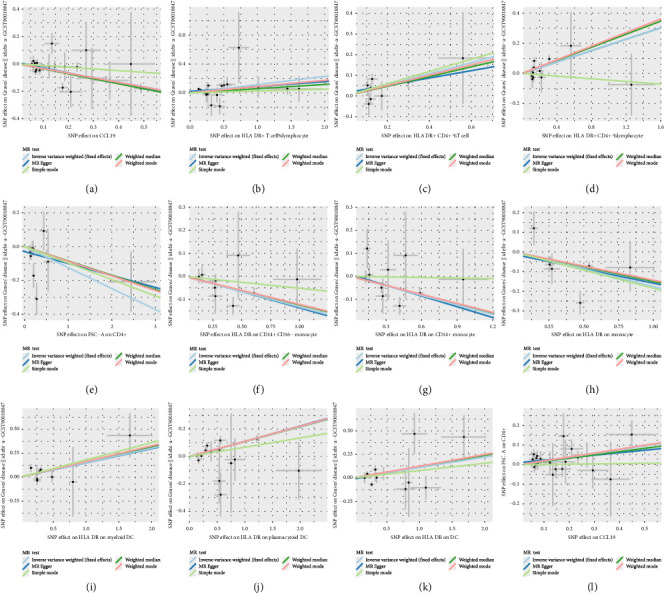
(a) Scatter plots of MR analyses between CCL19 on GD. (b–k) Scatter plots of MR analyses between circulating immune cells (HLA DR+ T cell% lymphocyte, HLA DR+ CD4+ %T cell, HLA DR+ CD4+ %lymphocyte, FSC-A on CD4+, HLA DR on CD14+ CD16− monocyte, HLA DR on CD14+ monocyte, HLA DR on monocyte, HLA DR on myeloid DC, HLA DR on plasmacytoid DC, HLA DR on DC) on GD. (l) Scatter plots of MR analyses between CCL19 on FSC-A on CD4+.

In the results with significant causal relationships that we focused on, no heterogeneity was observed among SNPs based on Cochrane's Q test (*p* > 0.05). Among the significant causal pairs identified by IVW, with the exception of the relationship between Transitional % B cells and GD, no evidence of horizontal pleiotropy was observed for other causal pairs, as indicated by the intercept from the MR-Egger analysis (*p* > 0.05). Additionally, the MR-PRESSO method did not detect any outliers. More detailed sensitivity analysis results are provided in Supporting Tables 9–12. The leave-one-out analysis further confirmed that no single SNP had a significant influence on the outcome (Supporting Figure 1). Furthermore, scatter and funnel plots excluded the possibility of potential outliers and horizontal pleiotropy (Figure 9 and Supporting Figure 2). The results of the MR-Steiger analysis confirmed the correctness of the directionality, with no evidence of reverse causality (Supporting Tables 4, 5, 7). These sensitivity analyses collectively excluded the influence of heterogeneity and horizontal pleiotropy, confirming the robustness of the results.

## 4. Discussion

In this study, we conducted two-sample bidirectional MR and mediation MR analyses to investigate the causal relationships between inflammatory cytokines/proteins, immune cells, and three thyroid diseases, and to determine whether immune cells act as mediators in the pathway between inflammatory factors/proteins and the thyroid diseases. Our findings indicate that CCL19 is negatively associated with GD: higher levels of CCL19 may be linked to a lower risk of GD. Additionally, CCL19 showed a positive association with FSC-A on CD4+ T cells, where elevated CCL19 levels correlated with a larger FSC-A (cell size) of CD4+ T cells. Furthermore, FSC-A on CD4+ was negatively associated with GD, suggesting that larger CD4+ T cells (higher FSC-A) are linked to a reduced risk of GD. These results imply that immune cells may serve as mediators in the pathway involving circulating inflammatory proteins and GD.

CCL19, also known as MIP-3 beta, is an immunostimulatory chemokine. Chemokines are a critical class of cytokines that primarily regulate the migration, localization, and activation of immune cells through binding to chemokine receptors on their surface, playing a pivotal role in immune responses, inflammatory reactions, and tissue repair. For example, Ye et al. found that CCL4 in the chemokine family increases the risk of stomatitis [29]. CCL19 exerts its effects by binding to its receptor CCR7. The CCL19-CCR7 signaling pathway is involved in various biological processes, such as lymph node homeostasis, T cell activation, immune tolerance, inflammatory responses, and cancer metastasis [30–35]. The disruption of immune tolerance can lead to autoimmune diseases, such as rheumatoid arthritis (RA) and systemic lupus erythematosus (SLE), where the immune system mistakenly attacks self-tissues. Animal studies have shown that CCR7-deficient mice are prone to developing systemic multiorgan autoimmunity [36]. Currently, research on the relationship between CCL19 and GD is limited. However, CCL19 has been widely studied in other autoimmune diseases, such as RA, SLE, and multiple sclerosis [37–39]. Studies have found that the upregulation of chemokines CCL19 and CCL21 and their receptor CCR7 is associated with the pathogenesis of RA. Elevated levels of CCL19 have been detected in the synovial tissue and serum of RA patients [37, 40, 41]. In SLE patients, higher serum CCL19 levels are linked to the production of autoantibodies, and CCL19 may contribute to SLE pathogenesis by disrupting B cell subset homeostasis [38]. In these autoimmune diseases, CCL19 often acts as a pathogenic inflammatory factor in the disease mechanisms. In contrast, our MR analysis suggests that higher circulating levels of CCL19 may be a protective factor against GD. This indicates that the balance of CCL19 levels plays a crucial role in autoimmune diseases. Higher levels of CCL19 may help maintain immune system balance, thereby preventing aberrant immune responses that lead to GD.

FSC-A in flow cytometry is primarily used to measure cell size. The FSC-A signal correlates with the intensity of light scattered as a cell passes through a laser beam; typically, the larger the cell, the more light it scatters, resulting in a higher FSC-A value. CD4+ T cells are a multifunctional subset of T cells in the immune system, primarily responsible for assisting and regulating immune responses. They play a critical role in the pathogenesis of autoimmune diseases [42]. Our study demonstrated a positive correlation between CCL19 and FSC-A of CD4+ T cells, suggesting that CCL19 may promote the activation or increase the size of CD4+ T cells. Immune cells typically undergo morphological changes, including an increase in cell volume, during the activation process [43, 44]. CCL19 can influence the activation process of CD4+ T cells through its receptor CCR7 [31, 45]. Activated CD4+ T cells usually exhibit a larger cell volume, leading to an enhanced FSC-A signal. Additionally, as a chemokine, CCL19 can stimulate the migration and tissue localization of CD4+ T cells [46, 47]. During this process, cells may undergo remodeling, affecting their size and morphology, which may be part of the cells' preparation for immune challenges. The negative correlation observed between FSC-A CD4+ T cells and GD suggests that maintaining an activated state or a larger volume of CD4+ T cells may have a protective effect against GD. This protective effect might be associated with the role of larger CD4+ T cells in maintaining a balanced immune response and immune tolerance, thereby reducing the risk of autoimmune attacks on the thyroid.

These associations suggest that the CD4+ T cell size (FSC-A) phenotype acts as a mediator between CCL19 and GD. CCL19 may influence the risk of GD by affecting the size and activation state of CD4+ T cells. Larger CD4+ T cells (higher FSC-A) may be associated with a balanced immune state that helps prevent aberrant immune attacks on the thyroid. Additionally, MR studies have found associations between circulating inflammatory proteins, immune cells, and oral diseases [29, 48], indicating that they may also be related to the inflammation-immune cell-disease axis, which echoes the results of our study. However, this finding provides only a plausible hypothesis, and further experimental studies are required to elucidate how CCL19 specifically regulates CD4+ T cell size and function, as well as how these changes impact the development of GD. If the regulatory role of CCL19 is confirmed, it could offer a novel therapeutic approach for GD, such as modulating CCL19 levels to influence CD4+ T cell function, thereby controlling or preventing the disease.

Our study has several strengths [1]: The summary statistics for inflammatory cytokines/proteins were derived from the largest and most recent GWAS, and there were no overlapping samples in our study [2]. Strict criteria were established to screen IVs, removing SNPs potentially associated with confounding factors affecting the outcome. Additionally, to enhance the reliability of our findings, we conducted sensitivity analyses, MR-Steiger analysis, and applied FDR correction to *p* values.

Our study also has some limitations [1]: GWAS data were sourced from a European population, and it remains uncertain whether the findings are generalizable to other populations and regions [2]. Due to limitations in the available GWAS data, we were unable to stratify study subjects by gender. This study may not fully capture the potential gender-specific impact of thyroid diseases on circulating inflammatory cytokine levels [3]. This MR study focused on inflammatory cytokines from circulation, and there may be differences between cytokine levels in thyroid tissue and circulation. Further research is warranted to explore the bidirectional causal relationship between inflammatory cytokines within thyroid tissue and thyroid diseases.

## 5. Conclusion

In this MR study, we identified a causal relationship between circulating inflammatory proteins and immune cells in relation to GD, with immune cells serving as intermediaries in the pathway between inflammatory proteins and GD risk. These findings have not only deepened our understanding of the immunopathogenic mechanisms underlying GD, but also provided a theoretical basis for exploring CCL19 and CD4+ T cell-targeted interventions as potential preventive or therapeutic strategies for GD, and laid a foundation for future studies to validate these findings and elucidate the detailed molecular mechanisms.

## Figures and Tables

**Table 1:** Mediation Mendelian randomization analyses of the causal effects among circulating inflammatory proteins, immune cells and GD.

Exposure	Mediator	Outcome	Beta (total effect)	Beta1	Beta2	Indirect effect (beta1 × beta2)	Mediated proportion (%)
CCL19	FSC-A on CD4+	GD	−0.333	0.191	−0.124	−0.024	7.20

## Data Availability

In this study, all data were obtained from publicly available sources, including the IEU Open GWAS project database (https://gwas.mrcieu.ac.uk/), the data.bris Research Data Repository (https://data.bris.ac.uk/data/dataset/3g3i5smgghp0s2uvm1doflkx9x), and the GWAS Catalog (https://www.ebi.ac.uk/gwas/), all of which are freely accessible.

## Supporting Information

Supporting Information 1 STROBE-MR-checklist: Strengthening the Reporting of Observational Studies in Epidemiology-Mendelian Randomization checklist.

Supporting Information 2 Supporting Figure 1: (A) Leave-one-out sensitivity analyses of MR analyses between CCL19 on GD. (B-K) Leave-one-out sensitivity analyses of MR analyses between circulating immune cells (HLA DR + T cell% lymphocyte, HLA DR + CD4+ %T cell, HLA DR + CD4+ %lymphocyte, FSC−A on CD4+, HLA DR on CD14+ CD16− monocyte, HLA DR on CD14+ monocyte, HLA DR on monocyte, HLA DR on myeloid DC, HLA DR on plasmacytoid DC, HLA DR on DC) on GD. (L) Leave-one-out sensitivity analyses of MR analyses between CCL19 on FSC−A on CD4+. Supporting Figure 2: (A) Funnel plots of MR analyses between CCL19 on GD. (B-K) Funnel plots of MR analyses between circulating immune cells (HLA DR + T cell% lymphocyte, HLA DR + CD4+ %T cell, HLA DR + CD4+ %lymphocyte, FSC−A on CD4+, HLA DR on CD14+ CD16− monocyte, HLA DR on CD14+ monocyte, HLA DR on monocyte, HLA DR on myeloid DC, HLA DR on plasmacytoid DC, HLA DR on DC) on GD. (L) Funnel plots of MR analyses between CCL19 on FSC−A on CD4+.

Supporting Information 3 Supporting Table 1: The SNPs information for circulating inflammatory cytokines/proteins that exhibit a significant association with thyroid diseases. Supporting Table 2: The SNPs information for immune cells that exhibit a significant association with thyroid diseases. Supporting Table 3: The SNPs information for CCL19 that exhibit a significant association with immune cells. Supporting Table 4: MR estimates for the association between circulating inflammatory cytokines/proteins on thyroid diseases. Supporting Table 5: MR estimates for the association between thyroid diseases on circulating inflammatory cytokines/proteins. Supporting Table 6: MR estimates for the association between 12 immune cells on GD. Supporting Table 7: MR estimates for the association between immune cells on GD. Supporting Table 8: MR estimates for the association between CCL19 on immune cells. Supporting Table 9: The heterogeneity, Horizontal pleiotropy and MR-PRESSO analysis between circulating inflammatory cytokines/proteins on thyroid diseases. Supporting Table 10: The heterogeneity, Horizontal pleiotropy and MR-PRESSO analysis between thyroid diseases on circulating inflammatory cytokines/proteins. Supporting Table 11: The heterogeneity, Horizontal pleiotropy and MR-PRESSO analysis between immune cells on GD. Supporting Table 12: The heterogeneity, Horizontal pleiotropy and MR-PRESSO analysis between CCL19 on immune cells.
