# Supplementary material for: The Inflammatory-Immune Axis in Thyroid Disease: A Mendelian Randomization Study
Source: Int J Endocrinol. 2025 Jul 21;2025:6644708. doi: 10.1155/ije/6644708 (PMC12303634; doi:10.1155/ije/6644708)
Supplement: Supporting Information 2 — Supporting Figure 1: (A) Leave-one-out sensitivity analyses of MR analyses between CCL19 on GD. (B-K) Leave-one-out sensitivity analyses of MR analyses between circulating immune cells (HLA DR + T cell% lymphocyte, HLA DR + CD4+ %T cell, HLA DR + CD4+ %lymphocyte, FSC−A on CD4+, HLA DR on CD14+ CD16− monocyte, HLA DR on CD14+ monocyte, HLA DR on monocyte, HLA DR on myeloid DC, HLA DR on plasmacytoid DC, HLA DR on DC) on GD. (L) Leave-one-out sensitivity analyses of MR analyses between CCL19 on FSC−A on CD4+. Supporting Figure 2: (A) Funnel plots of MR analyses between CCL19 on GD. (B-K) Funnel plots of MR analyses between circulating immune cells (HLA DR + T cell% lymphocyte, HLA DR + CD4+ %T cell, HLA DR + CD4+ %lymphocyte, FSC−A on CD4+, HLA DR on CD14+ CD16− monocyte, HLA DR on CD14+ monocyte, HLA DR on monocyte, HLA DR on myeloid DC, HLA DR on plasmacytoid DC, HLA DR on DC) on GD. (L) Funnel plots of MR analyses between CCL19 on FSC−A on CD4+. [file 6644708.f2.docx]

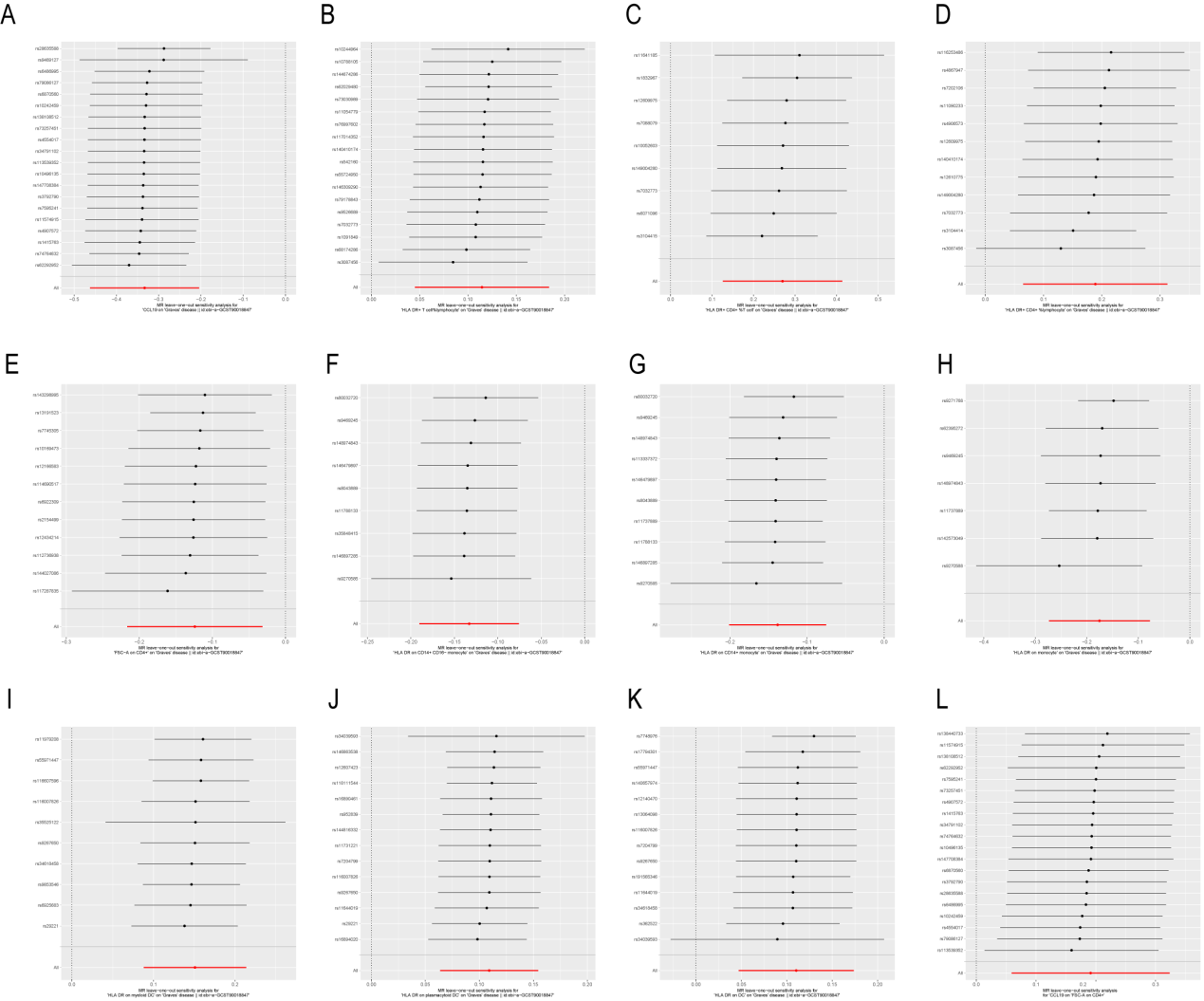


Supplemental Fig. 1

(A) Leave-one-out sensitivity analyses of MR analyses between CCL19 on GD.

(B-K) Leave-one-out sensitivity analyses of MR analyses between circulating immune cells (HLA DR+ T cell% lymphocyte, HLA DR+ CD4+ %T cell, HLA DR+ CD4+ %lymphocyte, FSC−A on CD4+, HLA DR on CD14+ CD16− monocyte, HLA DR on CD14+ monocyte, HLA DR on monocyte, HLA DR on myeloid DC, HLA DR on plasmacytoid DC, HLA DR on DC) on GD.

(L) Leave-one-out sensitivity analyses of MR analyses between CCL19 on FSC−A on CD4+.


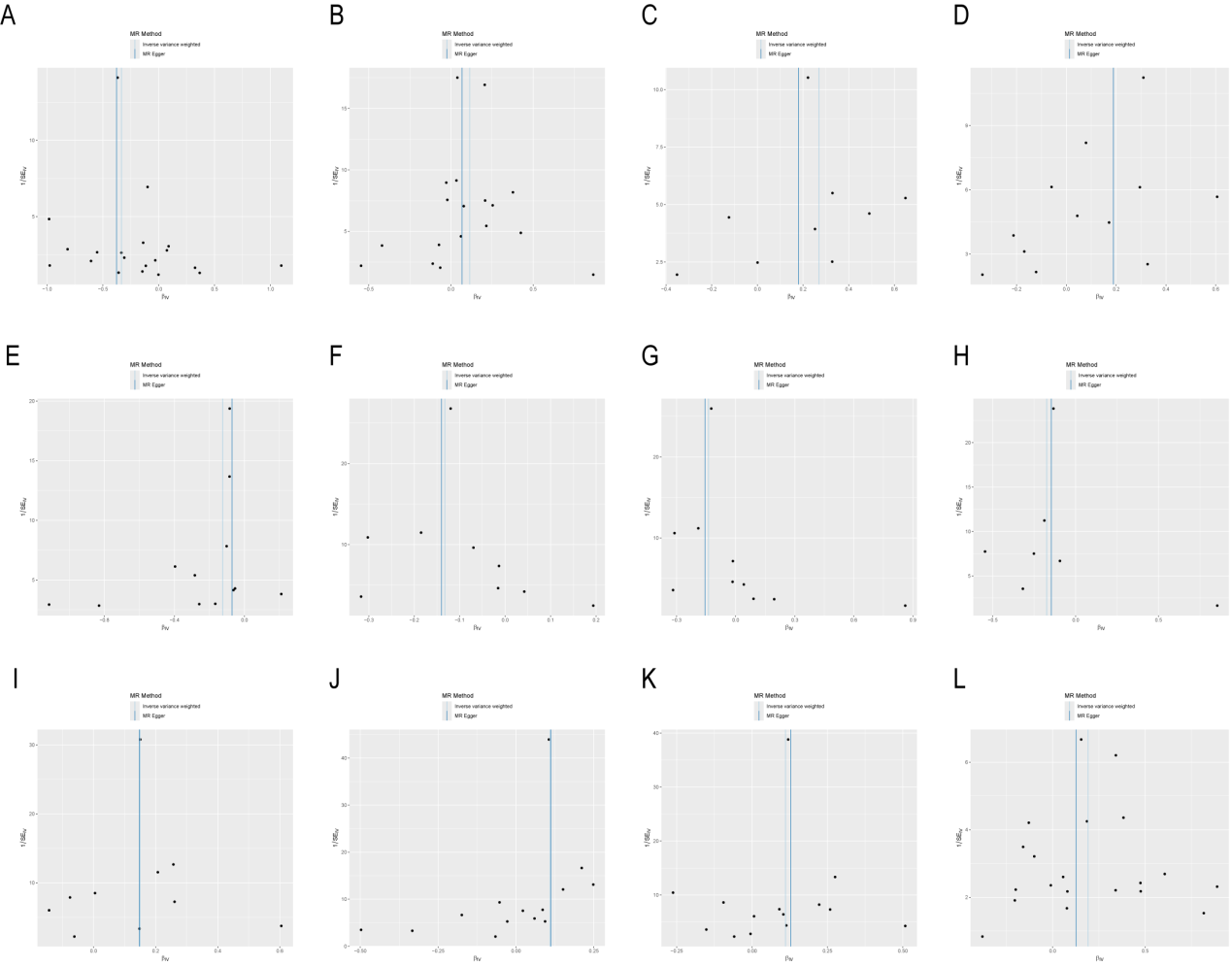


Supplemental Fig. 2

(A) Funnel plots of MR analyses between CCL19 on GD.

(B-K) Funnel plots of MR analyses between circulating immune cells (HLA DR+ T cell% lymphocyte, HLA DR+ CD4+ %T cell, HLA DR+ CD4+ %lymphocyte, FSC−A on CD4+, HLA DR on CD14+ CD16− monocyte, HLA DR on CD14+ monocyte, HLA DR on monocyte, HLA DR on myeloid DC, HLA DR on plasmacytoid DC, HLA DR on DC) on GD.

(L) Funnel plots of MR analyses between CCL19 on FSC−A on CD4+.
